# Algorithms for Hidden Markov Models Restricted to Occurrences of Regular Expressions

**DOI:** 10.3390/biology2041282

**Published:** 2013-11-08

**Authors:** Paula Tataru, Andreas Sand, Asger Hobolth, Thomas Mailund, Christian N. S. Pedersen

**Affiliations:** 1 Bioinformatics Research Centre, Aarhus University, C. F. Møllers Allé 8, DK-8000 Aarhus C, Denmark; E-Mails: paula@birc.au.dk (P.T.); asand@birc.au.dk (A.S.); hobolth@birc.au.dk (A.H.); mailund@birc.au.dk (T.M.); 2 Department of Computer Science, Aarhus University, Aabogade 34, DK-8200 Aarhus N, Denmark

**Keywords:** Hidden Markov Model, decoding, Viterbi, forward, algorithm

## Abstract

Hidden Markov Models (HMMs) are widely used probabilistic models, particularly for annotating sequential data with an underlying hidden structure. Patterns in the annotation are often more relevant to study than the hidden structure itself. A typical HMM analysis consists of annotating the observed data using a decoding algorithm and analyzing the annotation to study patterns of interest. For example, given an HMM modeling genes in DNA sequences, the focus is on occurrences of genes in the annotation. In this paper, we define a pattern through a regular expression and present a restriction of three classical algorithms to take the number of occurrences of the pattern in the hidden sequence into account. We present a new algorithm to compute the distribution of the number of pattern occurrences, and we extend the two most widely used existing decoding algorithms to employ information from this distribution. We show experimentally that the expectation of the distribution of the number of pattern occurrences gives a highly accurate estimate, while the typical procedure can be biased in the sense that the identified number of pattern occurrences does not correspond to the true number. We furthermore show that using this distribution in the decoding algorithms improves the predictive power of the model.

## 1. Introduction

A Hidden Markov Model (HMM) is a probabilistic model for sequential data with an underlying hidden structure. Because of their computational and analytical tractability, they are widely used especially in speech recognition [1,2,3], image processing [4] and in several applications in bioinformatics; e.g., modeling of proteins [5,6,7], sequence alignment [8,9,10], phylogenetic analysis [11,12] and identification of coding regions in genomes [13,14].

Patterns in the hidden structure are, however, often more relevant to study than the full hidden structure itself. When modeling proteins, one might be interested in neighboring secondary structures that differ, while for sequence alignments, the pattern could capture specific characteristics, such as long indels. In phylogenetic analysis, changes in the tree along the sequence are most relevant, while when investigating coding regions of DNA data, patterns corresponding to genes are the main focus.

Counting the number of occurrences of such patterns can be approached (as in the methods based on [15]) by making inferences from the prediction of a decoding algorithm; e.g., the Viterbi algorithm or the posterior-Viterbi algorithm. As we show in our experiments, this can give consistently biased estimates for the number of pattern occurrences. A more realistic method is presented in [16], where the distribution of the number of pattern occurrences is computed by means of Markov chain embedding. To our knowledge, this is the only study of patterns in the hidden sequence of HMMs. The problem of pattern finding in random sequences generated by simple models, such as Markov chains, has been intensively studied using the embedding technique [17,18,19].

We present a fundamentally different approach to compute the distribution of the number of pattern occurrences and show how it can be used to improve the prediction of the hidden structure. We use regular expressions as patterns and employ their deterministic finite automata to keep track of occurrences. The use of automata to describe occurrences of patterns in Markov sequences has been described previously in [18,20,21]. However, analyzing pattern occurrences in the hidden structure of HMMs by means of automata has not been done before. We introduce a new version of the forward algorithm, the restricted forward algorithm, which computes the likelihood of the data under the hidden sequence containing a specific number of pattern occurrences. This algorithm can be used to compute the occurrence number distribution. We furthermore introduce new versions of the two most widely used decoding algorithms, the Viterbi algorithm and the posterior-Viterbi algorithm, where the prediction is restricted to containing a certain number of occurrences, e.g., the expectation obtained from the distribution. We have implemented and tested the new algorithms and performed experiments that show that this approach improves both the estimate of the number of pattern occurrences and the prediction of the hidden structure.

The remainder of this paper is organized as follows: we start by introducing Hidden Markov Models and automata; we continue by presenting our restricted algorithms, which we then validate experimentally.

## 2. Methods

### 2.1. Hidden Markov Models

A Hidden Markov Model (HMM) [3] is a joint probability distribution over an observed sequence *y*_1:_*_T_* = *y*_1_*y*_2_…*y_T_* ∈ 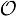
* and a hidden sequence *x*_1:_*_T_* = *x*_1_*x*_2_ … *x_T_* ∈ 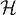
*, where 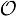 and 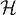 are finite alphabets of observables and hidden states, respectively. The hidden sequence is a realization of a Markov process that explains the hidden properties of the observed data. We can formally define an HMM as consisting of a finite alphabet of hidden states 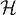 = {*h*_1_, *h*_2_, …, *h_N_*}, a finite alphabet of observables 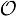 = {*o*_1_,*o*_2_, …, *o_M_*}, a vector Π = (*π_h_i__*)_1≤_*_i_*_≤_*_N_*, where *π_h_i__* = ℙ(*X*_1_ = *h_i_*) is the probability of the hidden sequence starting in state *h_i_*, a matrix *A* = {*a_h_i__*_,_*_h_j__*}_1≤_*_i_*_,_*_j_*_≤_*_N_*, where *a_h_i__*_,_*_h_j__* = ℙ(*X_t_* = *h_j_* | *X_t_*_−1_ = *h_i_*) is the probability of a transition from state *h_i_* to state *h_j_*, and a matrix $B={\left\{b_{h_{i},o_{j}}\right\}}_{1\leq i\leq N}^{1\leq j\leq M}$, where *b_h_i__*_,_*_o_j__* = ℙ(*Y_t_* = *o_j_* | *X_t_* = *h_i_*) is the probability of state *h_i_* emitting *o_j_*.

**Figure 1:**
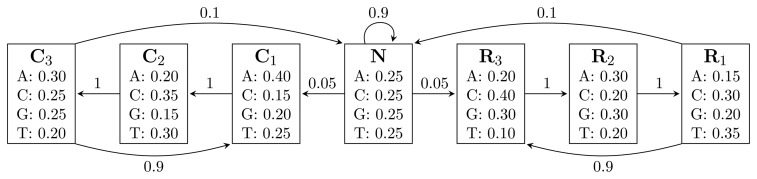
A Hidden Markov Model (HMM) for gene prediction. Each box represents a hidden state, and the numbers inside are the emission probabilities of each nucleotide. Numbers on arcs are transition probabilities between hidden states.

Figure 1 shows an HMM designed for gene finding. The observed sequence is a DNA sequence over the alphabet 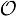 = {*A*, *C*, *G*, *T*}, while the hidden states encode if a position is in a non-coding area (*N*) or is part of a gene on the positive strand (*C*) or on the negative strand (*R*). The model incorporates the fact that nucleotides come in multiples of three within genes, where each nucleotide triplet codes for an amino acid. The set of hidden states is 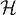 = {*N*} ∪ {*C_i_*, *R_i_*|1 ≤ *i* ≤ 3}. In practice, models used for gene finding are much more complex, but this model captures the essential aspects of a gene finder.

HMMs can be used to generate sequences of observables, but their main application is for analyzing an observed sequence, *y*_1:_*_T_*. The likelihood of a given observed sequence can be computed using the forward algorithm [3], while the Viterbi algorithm [3] and the posterior-Viterbi algorithm [22] are used for predicting a corresponding hidden sequence. All these algorithms run in 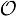 (*TN*^2^) time, using 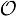 (*TN*) space.

#### 2.1.1. The Forward Algorithm

The forward algorithm [3] finds the probability of observing *y*_1:_*_T_* by summing the joint probability of the observed and hidden sequences for all possible sequences, *x*_1:_*_T_*. This is given by:

(1) $$\mathbb{P}(y_{1:T},x_{1:T})=\pi_{x_{1}}b_{x_{1},y_{1}}\prod_{t=2}^{T}a_{x_{t}-1,x_{t}}b_{x_{t},y_{t}}$$

(2) $$\mathbb{P}(y_{1:T})=\sum_{x_{1:T}}\mathbb{P}(y_{1:T},x_{1:T})$$

where Equation (1) is the multiplication of the probabilities of transitions and emissions, which explain observing *y*_1:_*_T_* with *x*_1:_*_T_* as the hidden sequence: the HMM starts in state *x*_1_ and emits *y*_1_ from *x*_1_, and for all *t* = 2, …, *T*, it makes a transition from state *x_t_*_−1_ to *x_t_* and emits *y_t_* from *x_t_*.

The forward algorithm finds Equation (2) by recursively filling up a table, *α*, with values *α_t_*(*x_t_*) = ℙ(*y*_1:_*_t_*, *x_t_*) = Σ*x*_1:_*_t_*_−1_ ℙ(*y*_1:_*_t_*, *x*_1:_*_t_*) being the probability of observing *y*_1:_*_t_* and being in state *x_t_* at time *t*. The recursion is:

(3) $$\begin{array}{ll} \alpha_{1}(h_{i}) & =\pi_{h_{i}}b_{h_{i},y_{1}}\\ \alpha_{t}(h_{i}) & =b_{h_{i},y_{t}}\underset{j}{\text{∑}}\alpha_{t-1}(h_{j})a_{h_{j},h_{i}} \end{array}$$

and, finally, ℙ(*y*_1:_*_T_*) = Σ*_i_ α_T_*(*h_i_*).

#### 2.1.2. The Viterbi Algorithm

The Viterbi algorithm [3] finds the sequence of hidden states, *x*_1:_*_T_*, that maximizes the joint probability of the observed and hidden sequences (1). It uses the same type of approach as the forward algorithm: a new table, *ω*, is defined by *ω_t_*(*x_t_*) = max*_x_*__1:_*t*_−1__ {ℙ(*y*_1:_*_t_*, *x*_1:_*_t_*)}, the probability of a most likely decoding ending in *x_t_* at time *t*, having observed *y*_1:_*_t_*. This can be obtained as follows:

(4) $$\begin{array}{ll} \omega_{1}(h_{i}) & =\pi_{h_{i}}b_{h_{i},y_{1}}\\ \omega_{t}(h_{i}) & =b_{h_{i},y_{t}}\cdot \underset{j}{\max}\{\omega_{t-1}(h_{j})a_{h_{j},h_{i}}\} \end{array}$$

After computing *ω*, a most likely sequence of hidden states is retrieved by backtracking through the table, starting in entry argmax*_h_i__* {*ω_T_*(*h_i_*)}.

#### 2.1.3. The Posterior Decoding Algorithm

The posterior deco ding [3] of an observed sequence is an alternative to the prediction given by the Viterbi algorithm. While the Viterbi algorithm computes the decoding with the highest joint probability, the posterior decoding computes a sequence of hidden states, *x*_1:_*_T_*, such that *x_t_* = argmax*_h_i__* {*γ_t_*(*h_i_*)} has the highest posterior probability *γ_t_*(*h_i_*) = ℙ(*h_i_* | *y*_1:_*_T_*). If we let *β_t_*(*h_i_*) = ℙ(*y_t_*_+1:_*_T_* | *h_i_*), we have:

(5) $$\begin{array}{ll} \gamma_{t}(h_{i}) & =\frac{\mathbb{P}(h_{i},y_{1:t})\mathbb{P}(y_{t+1:T}|h_{i})}{\mathbb{P}(y_{1:T})}\\ & =\frac{\alpha_{t}(h_{i})\beta_{t}(h_{i})}{\sum_{j}\alpha_{T}(h_{j})} \end{array}$$

The backward algorithm [3] gives *β_t_*(*h_i_*) by using a recursion similar to Equation (3). Thus, to compute the posterior decoding, we first fill out *α_t_*(*h_i_*) and *β_t_*(*h_i_*) for all *t* and *i* and then compute the decoding by *x_t_* = argmax*_h_i__* {*γ_t_*(*h_i_*)}.

#### 2.1.4. The Posterior-Viterbi Algorithm

The posterior decoding algorithm often computes decodings that are very accurate locally, but it may return syntactically incorrect decodings; *i.e.*, decodings with transitions that have a probability of zero. The posterior-Viterbi algorithm [22] corrects for this by computing a syntactically correct decoding $x_{1:T}^{*}={\mathrm{argmax}}_{x_{1:T}\in A_{p}}\left\{\prod_{t=1}^{T}\gamma_{i}(x_{i})\right\}$ with the highest posterior probability, where *A_p_* is the set of syntactically correct decodings. To compute this, a new table, *γ˜*, is defined by ${\tilde{\gamma }}_{t}(x_{t})=\max_{x_{1:t-1}\in A_{p}}\left\{\prod_{i=1}^{t}\gamma_{t}(x_{t})\right\}$, the maximum posterior probability of a decoding from *A_p_* ending in *x_t_* at time *t*. The table is filled using the recursion:

(6) $$\begin{array}{ll} {\tilde{\gamma }}_{1}(h_{i}) & =\gamma_{1}(h_{i})\\ {\tilde{\gamma }}_{t}(h_{i}) & =\gamma_{t}(h_{i})\cdot \underset{\left\{j:a_{h_{j},h_{i}}>0\right\}}{\max}\{{\tilde{\gamma }}_{t-1}(h_{j})\} \end{array}$$

After computing *γ˜*, a decoding from *A_p_* with the highest posterior probability is retrieved by backtracking through *γ˜* from entry argmax*_h_i__* {*γ˜_T_*(*h_i_*)}. We note that, provided that the posterior decoding algorithm returns a decoding from *A_p_*, the posterior-Viterbi algorithm will return the same decoding.

### 2.2. Automata

In this paper, we are interested in patterns over the hidden alphabet 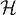 = {*h*_1_, *h*_2_, …, *h_N_*} of an HMM. Let *r* be a regular expression over 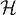, and let *FA*_
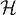
_(*r*) = (*Q*, 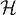, *q*_0_, *A*, *δ*) [23] be the deterministic finite automaton (DFA) that recognizes the language described by (*h*_1_ ∣ *h*_2_ ∣ … ∣ *h_N_*)*(*r*), where *Q* is the finite set of states, *q*_0_ ∈ *Q* is the initial state, *A* ⊆ *Q* is the set of accepting states and *δ* : *Q* × 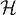 → *Q* is the transition function. *FA*_
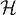
_(*r*) accepts any string that has *r* as a suffix. We construct the DFA *EA*_
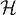
_(*r*) = (*Q*, 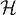, *q*_0_, *A*, *δ_E_*) as an extension of *FA*_
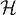
_(*r*), where *δ_E_* is defined by:

(7) $$\begin{array}{lll} \forall q\in Q\backslash A, & \forall h\in H, & \delta_{E}(q,h)=\delta (q,h)\\ \forall q\in A, & \forall h\in H, & \delta_{E}(q,h)=\delta (q_{0},h) \end{array}$$

**Figure 2:**
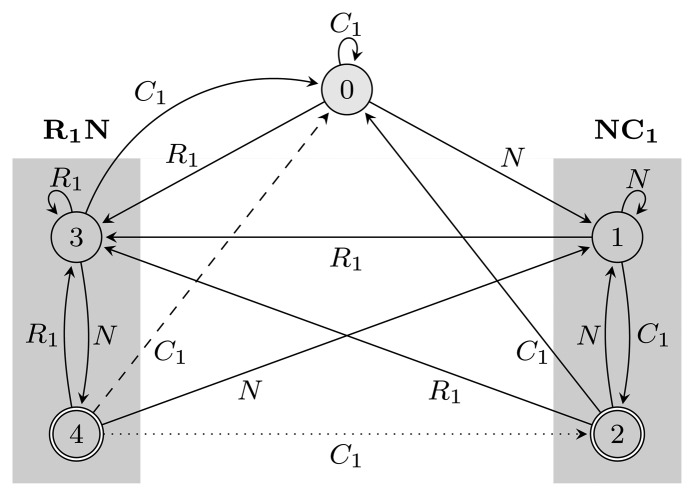
Two automata, *FA*_
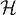
_(*r*) and *EA*_
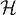
_(*r*), for the pattern *r* = (*NC*_1_) ∣ (*R*_1_*N*), 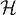 = {*N*} ∪ {*C_i_*, *R_i_* ∣ 1 ≤ *i* ≤ 3}, *Q* = {0, 1, 2, 3, 4}, *q*_0_ = 0, and *A* = {2, 4}. States 1, 2 and 3, 4 are used for matching sequences ending with *NC*_1_ and *R*_1_*N*, respectively, as marked with gray boxes. The two automata differ only with respect to transitions from accepting states: the dotted transition belongs to *FA*_
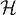
_(*r*) and the dashed one to *EA*_
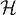
_(*r*). For clarity, the figure lacks transitions going from all states to state 0 using *C*_2_, *C*_3_, *R*_2_ and *R*_3_.

Essentially, *EA*_
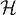
_(*r*) restarts every time it reaches an accepting state. Figure 2 shows *FA*_
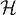
_(*r*) and *EA*_
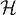
_(*r*) for *r* = (*NC*_1_) ∣ (*R*_1_*N*) with the hidden alphabet 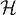 = {*N*} ∪ {*C_i_*, *R_i_* ∣ 1 ≤ *i* ≤ 3} of the HMM from Figure 2. Both automata have *Q* = {0, 1, 2, 3, 4}, *q*_0_ = 0, and *A* = {2, 4}. State 1 marks the beginning of *NC*_1_, while state 3 corresponds to the beginning of *R*_1_*N*. State 2 accepts *NC*_1_, and state 4 accepts *R*_1_*N*. As *C*_2_, *C*_3_, *R*_2_ and *R*_3_ are not part of *r*, using them, the automaton restarts by transitioning to state 0 from all states. We left these transitions out of the figure for clarity. The main difference between *FA*_
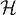
_(*r*) and *EA*_
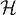
_(*r*) is that they correspond to overlapping and non-overlapping occurrences, respectively. For example, for the input string, *R*_1_*NC*_1_, *FA*_
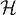
_(*r*) first finds *R*_1_*N* using state 4, from which it transitions to state 2 and matches *NC*_1_. However, after *EA*_
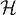
_(*r*) recognizes *R*_1_*N*, it transitions back to state 0, not matching *NC*_1_. The algorithms we provide are independent of which of the two automata is used, and therefore, all that remains is to switch between them when needed. In our implementation, we used an automata library for Java [24] to obtain *FA*_
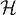
_(*r*), which we then converted to *EA*_
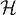
_(*r*).

## 3. Results and Discussion

Consider an HMM as defined previously, and let *r* be a regular expression over the alphabet of hidden states, 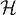. We present a modified version of the forward algorithm to compute the distribution of the number of occurrences of *r*, which we then use in an adaptation of the Viterbi algorithm and the posterior-Viterbi algorithm to obtain an improved prediction.

### 3.1. The Restricted Forward Algorithm

Let *O_r_*(*x*_1:_*_T_*) be the number of matches of *r* in *x*_1:_*_T_*. We wish to estimate *O_r_* by using its probability distribution. We do this by running the HMM and *FA*_
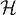
_(*r*) in parallel. Let *FA*_
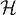
_(*r*)*_t_* be the state in which the automaton is after *t* transitions, and define *α̂_t_*(*x_t_*, *k*, *q*) = Σ_{_*_x_*__1:_*t*_−1_:_*_O_r__*_(_*_x_*__1:_*t*) =_*_k_*_}_ ℙ(*y*_1:_*_t_*, *x*_1:_*_t_*, *FA*_
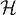
_(*r*)*_t_* = *q*) to be the entries of a new table, *α̂*, where *k* = 0, …, *m* and *m* ≤ *T* is the maximum number of pattern occurrences in a hidden sequence of length *T*. The table entries are the probabilities of having observed *y*_1:_*_t_*, being in hidden state *x_t_* and automaton state *q* at time *t* and having seen *k* occurrences of the pattern, corresponding to having visited accepting states *k* times. Letting *δ*^−1^(*q*, *h_i_*) = {*q′* ∣ *δ*(*q′*, *h_i_*) = *q*} be the automaton states from which a transition to *q* exists, using hidden state *h_i_* and 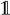 being the indicator function, mapping a Boolean expression to one if it is satisfied and to zero otherwise, we have that:

(8) $${\hat{\alpha }}_{t}(x_{t},k,q)=\sum_{\underset{O_{r}(x_{1:t-1})=k-1(q\in A)}{x_{1:t-1}:}}\mathbb{P}(y_{1:t},x_{1:t},FA_{H}{\left(r\right)}_{t}=q)$$

(9) $$\begin{array}{ll} \mathbb{P}(y_{1:t},x_{1:t}FA_{H}{\left(r\right)}_{t}=q) & =\underset{q^{\prime }\in \delta^{-1}(q,x_{t})}{\text{∑}}\mathbb{P}(y_{1:t},x_{1:t},FA_{H}{\left(r\right)}_{t-1}=q^{\prime })\\ & =b_{x_{t},y_{t}}a_{x_{t-1},x_{t}}\underset{q^{\prime }\in \delta^{-1}(q,x_{t})}{\text{∑}}\mathbb{P}(y_{1:t-1},x_{1:t-1},FA_{H}{\left(r\right)}_{t-1}=q^{\prime }) \end{array}$$

Using Equations (8) and (9), the recursion for *α̂* becomes:

(10) $$\begin{array}{ll} {\hat{\alpha }}_{1}(h_{i},k,q) & =\pi_{h_{i}}b_{h_{i},y_{1}}1(q_{0}\in \delta^{-1}(q,h_{i}))\cdot \{\begin{array}{ll} 1(q\notin A) & \text{if}\;k=0\\ 1(q\in A) & \text{if}\;k=1\\ 0 & \text{otherwise} \end{array}\\ {\hat{\alpha }}_{t}(h_{i},k,q) & =b_{h_{i},y_{t}}\underset{j}{\text{∑}}a_{h_{j},h_{i}}\underset{q^{\prime }\in \delta^{-1}(q,h_{i})}{\text{∑}}{\hat{\alpha }}_{t-1}(h_{j},k-1(q\in A),q^{\prime }) \end{array}$$

These probabilities now allow for the evaluation of the distribution of the number of occurrences, conditioned on the observed data:

(11) $$\begin{array}{ll} \mathbb{P}(k\;\text{occurrences of}\;r|y_{1:T}) & =\frac{\mathbb{P}(k\;\text{occurrences of}\;r,y_{1:T})}{\mathbb{P}(y_{1:T})}\\ & =\frac{1}{\mathbb{P}(y_{1:T})}\underset{i,q}{\text{∑}}{\hat{\alpha }}_{T}(h_{i},k,q) \end{array}$$

from which the expectation can be computed. The likelihood of the data can be obtained from either the forward or restricted forward algorithm, ℙ(*y*_1:_*_T_*) = Σ*_i_ α*(*h_i_*) = Σ*_i_*_,_*_k_*_,_*_q_α̂_T_*(*h_i_*, *k*, *q*).

The *α̂* table has *T* · *N* · *m* · |*Q*| entries, and the computation of each requires 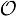 (*N*|*Q*|), leading to a 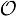 (*TN*^2^*m*|*Q*|^2^) running time and a space consumption of 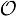 (*TNm*|*Q*|). In practice, both time and space consumption can be reduced. The restricted forward algorithm can be run for values of *k* that are gradually increasing up to *k_max_* for which ℙ(at most *k_max_* occurrences of *r* ∣ *y*_1:_*_T_*) is greater than, e.g., 99.99%. This *k_max_* is generally significantly less than *m*, while the expectation of the number of matches of *r* can be reliably calculated from this truncated distribution. The space consumption can be reduced to 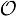 (*N*|*Q*|), because the calculation at time *t* for a specific value, *k*, depends only on the results at time *t* − 1 for *k* and *k* − 1.

### 3.2. Restricted Decoding Algorithms

The aim of the restricted decoding algorithms is to obtain a sequence of hidden states, *x*_1:_*_T_*, for which *O_r_*(*x*_1:_*_T_*) ∈ [*l*, *u*], where *l* and *u* are set to, for example, the expected number of occurrences, which can be calculated from the distribution. The restricted decoding algorithms are built in the same way as the restricted forward was obtained: a new table is defined, which is filled using a simple recursion. The evaluation of the table is followed by backtracking to obtain a sequence of hidden states, which contains between *l* and *u* occurrences of the pattern. The two restricted decoding algorithms use 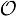 (*TNu*|*Q*|) space and 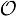 (*TN*^2^*u*|*Q*|^2^) time.

The entries in the table for the restricted Viterbi algorithm contain the probability of a most likely decoding containing *k* pattern occurrences, ending in state *x_t_* and automaton state *q* at time *t* and having observed *y*_1:_*_t_*, *ω̂_t_*(*x_t_*, *k*, *q*) = max_{_*_x_*__1:_*t*_−1_:_*_O_r__*_(_*_x_*__1:_*t*) =_*_k_*_}_ {ℙ(*y*_1:_*_t_*, *x*_1:_*_t_*, *FA*_
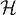
_(*r*)*_t_* = *q*)} with *k* = 0, …, *u*. The corresponding recursion is:

(12) $$\begin{array}{ll} {\hat{\omega }}_{1}(h_{i},k,q) & =\pi_{h_{i}}b_{h_{i},y_{1}}1(q_{0}\in \delta^{-1}(q,h_{i}))\cdot \{\begin{array}{ll} 1(q\notin A) & \text{if}\;k=0\\ 1(q\in A) & \text{if}\;k=1\\ 0 & \text{otherwise} \end{array}\\ {\hat{\omega }}_{t}(h_{i},k,q) & =b_{h_{i},y_{t}}\cdot \underset{j}{\max}\left\{a_{h_{j},h_{i}}\cdot \underset{q^{\prime }\in \delta^{-1}(q,h_{i})}{\max}\{{\hat{\omega }}_{t-1}(h_{j},k-1(q\in A),q^{\prime })\}\right\} \end{array}$$

and the backtracking starts in entry argmax*_i_*_,_*_k_*_∈[_*_l_*_,_*_u_*_],_*_q_* {*ω̂_T_*(*h_i_*, *k*, *q*)}.

For the restricted posterior-Viterbi algorithm, we compute the highest posterior probability of a decoding from *A_p_* containing *k* pattern occurrences, ending in state *x_t_* and automaton state *q* at time *t* and having observed *y*_1:_*_t_*, *γ̂_t_*(*x_t_*, *k*, *q*) = max_{_*_x_*__1:_*t*_−1_:_*_O_r__*_(_*_x_*__1:_*t*) =_*_k_*_}_ {ℙ(*x*_1:_*_t_*, *FA*_
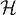
_(*r*)*_t_* = *q* ∣ *y*_1:_*_T_*)}. We have:

(13) $$\begin{array}{ll} {\hat{\gamma }}_{1}(h_{i},k,q) & =\gamma_{1}(h_{i})1(q_{0}\in \delta^{-1}(q,h_{i}))\cdot \{\begin{array}{ll} 1(q\notin A) & \text{if}\;k=0\\ 1(q\in A) & \text{if}\;k=1\\ 0 & \text{otherwise} \end{array}\\ {\hat{\gamma }}_{t}(h_{i},k,q) & =\gamma_{t}(h_{i})\cdot \underset{\left\{h_{j}:a_{h_{j},h_{i}}>0\right\}}{\max}\left\{\underset{q^{\prime }\in \delta^{-1}(q,h_{i})}{\max}\{{\hat{\gamma }}_{t-1}(h_{j},k-1(q\in A),q^{\prime })\}\right\} \end{array}$$

The backtracking starts in entry argmax*_i_*_,_*_k_*_∈[_*_l_*_,_*_u_*_],_*_q_* {*γ̂_T_*(*h_i_*, *k*, *q*)}.

### 3.3. Experimental Results on Simulated Data

We implemented the algorithms in Java, validated and evaluated their performance experimentally as follows: We first generated a test set consisting of 500 pairs of observed and hidden sequences for each length *L* = 500, 525, …, 1,500 from the gene finder in Figure 2. As the HMM is used for finding genes, an obvious choice of pattern is *r* = (*NC*_1_) ∣ (*R*_1_*N*), corresponding to the start of a gene. For each of the sequences, we estimated the number of overlapping pattern occurrences with the expected number of pattern occurrences, computed using the restricted forward algorithm, which we then used to run the restricted decoding algorithms. We also computed the prediction given by the two unrestricted decoding algorithms for comparison.

**Figure 3:**
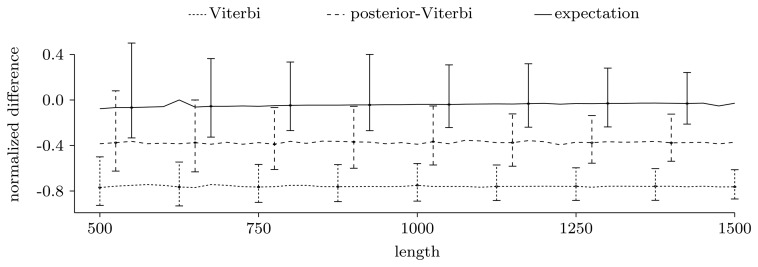
Normalized difference, $\frac{\text{estimate}}{\text{true value}}-1$, between the true number of pattern occurrences, the number given by the two unrestricted decoding algorithms and the expected number of pattern occurrences computed using the restricted forward algorithm. For each sequence length, we show the median of the normalized differences in the 500 experiments, together with the 0.025 and 0.975 quantiles, given as error bars.

#### 3.3.1. Counting Pattern Occurrences

Figure 3 shows the power of the restricted forward algorithm and the two unrestricted decoding algorithms to recover the true number of pattern occurrences. For each given length, we computed the normalized difference, $\frac{\text{estimate}}{\text{true value}}-1$, and plotted the median over the 500 sequences together with the 0.025 and 0.975 quantiles, given as error bars. As Figure 3 shows, the expectation gives a very good estimate. The decoding algorithms' performances are significantly lower, and they always give underestimates. This may be partly due to the model structure, where transitions to and from coding regions have low probability, leading to few, but long, genes.

#### 3.3.2. Quality of Predictions

We compared the predictive power of the two decoding algorithms in the original and restricted versions, using the expectation for the number of pattern occurrences. For each length in the test set, we measured the quality of each method, both at the nucleotide and gene level, following the analysis in [25]. Because the measures we use require binary data, we first converted the true hidden sequence and decoding to binary data containing non-coding areas and genes, by first considering the genes on the reverse strand as non-coding and calculating the measures for the genes on the direct strand, and *vice versa*. The final plotted measures are the averages obtained from the two conversions.

##### Nucleotide level

To investigate the quality at the nucleotide level, we compared the decoding and the true hidden state position by position. Each position can be classified as a true positive (predicted as part of a gene when it was part of a gene), true negative (predicted as non-coding when it was non-coding), false positive (predicted as part of a gene when it was non-coding) and false negative (predicted as non-coding when it was part of a gene). Using the total number of true positives (*tp*), true negatives (*tn*), false positives (*fp*) and false negatives (*fn*), we calculated the sensitivity, specificity and Matthew's correlation coefficient (MCC):

(14) $$\text{sens}=\frac{tp}{tp+fn}$$

(15) $$\text{spec}=\frac{tn}{tn+fp}$$

(16) $$\text{mcc}=\frac{tp\cdot tn-fp\cdot fn}{\sqrt{\left(tp+fp)\cdot (tp+fn)\cdot (tn+fp)\cdot (tn+fn\right)}}$$

Sensitivity and specificity are always between zero and one and relate to how well the algorithms are able to find genes (true positives) and non-coding regions (true negatives), respectively. MCC reflects the overall correctness and lies between −1 and 1, where 1 represents perfect prediction.

##### Gene level

**Figure 4:**
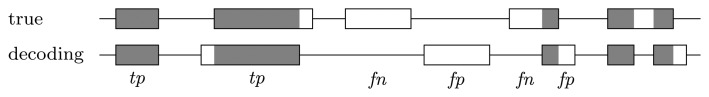
Error types at the gene level. A predicted gene is considered one true positive if it overlaps with at least 50% of a true gene and one false positive if there is no true gene with which it overlaps by at least 50%. Each true gene for which there is no predicted gene that overlaps by at least 50% counts as one false negative. True genes that are covered more than 50% by predicted genes, but for which there is no single predicted gene that covers a minimum of 50% are disregarded.

When looking at the decoding position by position, genes that are predicted correctly do not contribute to the measures in an equal manner, but rather, the longer the gene, the more contribution it brings. However, it is interesting how well the genes are recovered, independent of how long they are. To measure this, we consider a predicted gene as one true positive if it overlaps by at least 50% of a true gene and as one false positive if there is no true gene with which it overlaps by at least 50%. Each true gene for which there is no predicted gene that overlaps by at least 50% counts as one false negative; see Figure 4. In this context, true negatives, *i.e.*, the areas where there was no gene and no gene was predicted, are not considered, as they are not informative. The final measures are the recall, precision and F-score:

(17) $$\text{rec}=\frac{tp}{tp+fn}$$

(18) $$\text{prec}=\frac{tp}{tp+fp}$$

(19) $$f-\text{score}=2\cdot \frac{\text{rec}\cdot \text{prec}}{\text{rec}+\text{prec}}$$

(20) $$\begin{array}{ll} & =\frac{2\cdot tp}{2\cdot tp+fn+fp} \end{array}$$

They are all between zero and one and reflect how well the true genes have been recovered. The recall gives the fraction of true genes that have been found, while the precision gives the fraction of the predicted genes that are true genes. The F-score is the harmonic mean of the two.

**Figure 5:**
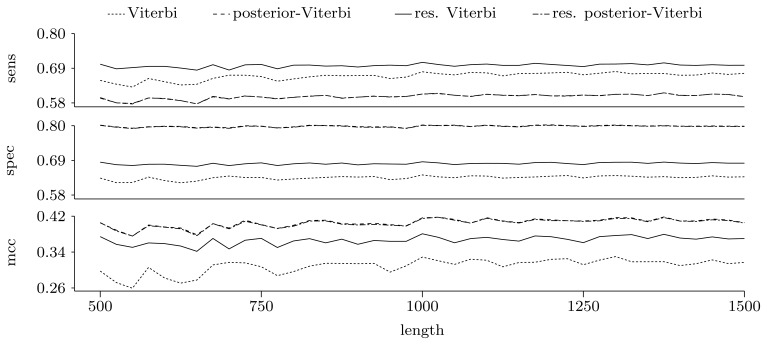
Prediction quality at the nucleotide level given by average sensitivity, specificity and Matthew's correlation coefficient (MCC) for the decoding algorithms. We ran the restricted decoding algorithms using the expectation calculated from the distribution returned by the restricted forward algorithm. The plots show a zoom-in of the three measures. As both sensitivity and specificity are between zero and one, the Y-axes in these two plots have the same scale.

**Figure 6:**
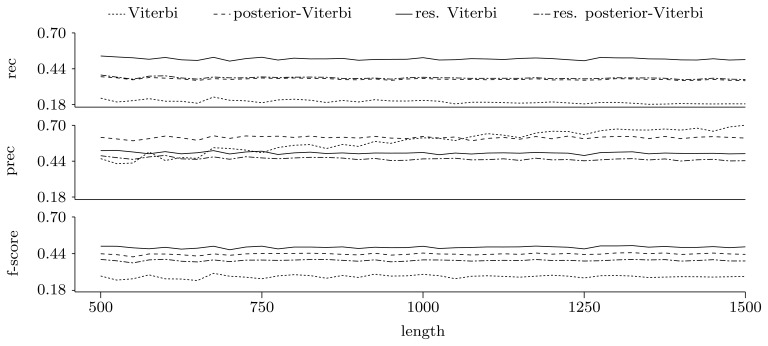
Prediction quality at the gene level given by average recall, precision and F-score for the decoding algorithms. We ran the restricted decoding algorithms using the expectation calculated from the distribution returned by the restricted forward algorithm. The plots show a zoom-in of the three measures with the same scale on the Y axes.

Figures 5 and 6 show the quality of predictions at the nucleotide and gene level, respectively. When comparing the Viterbi algorithm with the restricted Viterbi algorithm, it is clear that the restriction brings a great improvement to the prediction, as the restricted version has an increased power in all measures considered, with the exception of precision, where the Viterbi algorithm shows a tendency of increased precision with sequence length. However, when comparing the posterior-Viterbi algorithm with its restricted version, it is apparent that the restricted version does as good at the nucleotide level, but it performs worse at the gene level. By inspecting the predictions of the two methods, it was clear that the restricted posterior-Viterbi obtained an increased number of genes dictated by the expectation by just fractioning the genes predicted by the posterior-Viterbi. We believe this happens because the posterior-Viterbi algorithm finds the best local decoding, and therefore, adding global information, such as the total number of genes in the prediction, does not aid in the decoding. On the other hand, as the Viterbi algorithm finds the best global decoding, using the extra information results in a significant improvement of the prediction. Overall, at the nucleotide level, the posterior-Viterbi shows the best performance, while at the gene level, the restricted Viterbi has the highest quality. One might expect this, given the nature of the algorithms.

Apart from these experiments, we also ran the algorithms on the annotated *E. coli* genome (GenBank accession number U00096). In this set of experiments, we split the genome into sequences of a length of approximately 10,000, for which we ran the algorithms as previously described. We found the same trends as in the performance for simulated data (results not shown). When using the *E. coli* data, we also recorded the running time of the algorithms, and we found that the restricted algorithms are about 45 times slower than the standard algorithms, which is faster than the worst case scenario, which would lead to a *k* · |*Q*|^2^ = 7 · 5^2^ = 175 slowdown, as the average expectation of the number of patterns per sequence was *k* = 7.

## 4. Conclusions

We have introduced three novel algorithms that efficiently combine the theory of Hidden Markov Models with automata and pattern matching to recover pattern occurrences in the hidden sequence. First, we computed the distribution of the number of pattern occurrences by using an algorithm similar to the forward algorithm. This problem has been treated in [16] by means of Markov chain embedding, using simple finite sets of strings as patterns. Our method is, however, more general, as it allows the use of regular expressions.

From the occurrence number distribution, we calculated the expected number of pattern matches, which estimated the true number of occurrences with high precision. We then used the distribution to alter the prediction given by the two most widely used decoding algorithms: the Viterbi algorithm and the posterior-Viterbi algorithm. We have shown that in the case of the Viterbi algorithm, which finds the best global prediction, using the expected number of pattern occurrences greatly improves the prediction, both at the nucleotide and gene level. However, in the case of the posterior-Viterbi algorithm, which finds the best local prediction, such an addition only fragments the predicted genes, leading to a poorer prediction. Overall, deciding which algorithm is best depends on the final measure used, but as our focus was on finding genes, we conclude that the restricted Viterbi algorithm showed the best result.

As the distribution obtained from the restricted forward algorithm facilitates the calculation of the distribution of the waiting time until the occurrence of the *k*^th^ pattern match, the restricted Viterbi algorithm could potentially be further extended to incorporate this distribution while calculating the joint probability of observed and hidden sequences. Weighted transducers [26] are sequence modeling tools similar to HMMs, and analyzing patterns in the hidden sequence can potentially also be done by composition of the transducers, which describe the HMM and the automaton.

Our method can presumably be used with already existing HMMs to improve their prediction, by using patterns that reflect the problems studied using the HMMs. For example, in [13], an HMM is used for finding frameshifts in coding regions. In this situation, the pattern would capture the sequence of hidden states that corresponds to a frameshift. In HMMs used for phylogenetic analysis [11,12], the hidden states represent trees, and an event of interest is a shift in the tree. A pattern capturing a change in the hidden state could thus aid in improving the prediction. In HMMs built for probabilistic alignments [10], the pattern could capture the start of an indel, and our method could potentially aid in finding a more accurate indel rate estimate. There is therefore a substantial potential in the presented method to successfully improve the power of HMMs.
